# Daratumumab, Lenalidomide, and Dexamethasone Versus Bortezomib, Lenalidomide, and Dexamethasone in Transplant‐Ineligible Newly Diagnosed Multiple Myeloma: A Systematic Literature Review and Meta‐Analysis

**DOI:** 10.1002/hon.70061

**Published:** 2025-04-10

**Authors:** Lucio N. Gordan, Rohan Medhekar, Alex Z. Fu, Mostafa Shokoohi, Abril Oliva Ramirez, Nicolle Bonar, Bao‐Ngoc Nguyen, Michaela Spence, Rebecca McTavish, Tim Disher, Santosh Gautam, Niodita Gupta‐Werner, Shuchita Kaila, Anjan J. Patel

**Affiliations:** ^1^ Florida Cancer Specialists & Research Institute Gainesville Florida USA; ^2^ Real World Value and Evidence Oncology Janssen Scientific Affairs LLC Horsham Pennsylvania USA; ^3^ Real World Analytics Janssen Scientific Affairs LLC Titusville New Jersey USA; ^4^ Georgetown University Medical Center Washington District of Columbia USA; ^5^ Value & Evidence Division Marketing and Market Access EVERSANA Burlington Canada

**Keywords:** DRd, first‐line treatment, meta‐analysis, multiple myeloma, systematic review, transplant ineligible, VRd

## Abstract

Daratumumab in combination with lenalidomide and dexamethasone (DRd) and bortezomib in combination with lenalidomide and dexamethasone (VRd) are guideline‐recommended preferred regimens for initial treatment of transplant‐ineligible (TIE) patients with newly diagnosed multiple myeloma (NDMM). This study aimed to systematically identify evidence on the clinical effectiveness of DRd and VRd as first‐line treatments for patients with TIE NDMM and to conduct a meta‐analysis. Ovid MEDLINE, Embase, and Cochrane Library were searched from January 2019 to June 2023, along with key congresses from January 2018 to June 2023. Bibliographies of relevant systematic literature reviews (SLR) were hand‐searched. Randomized controlled trials and appropriately adjusted non‐randomized studies comparing DRd versus VRd as first‐line treatment for TIE NDMM were included. Overall, five records from three unique studies were identified. The fixed‐effects meta‐analysis showed a lower risk of disease progression or death with DRd versus VRd using the naïve approach (hazard ratio [HR]: 0.60; 95% confidence interval [CI]: 0.46, 0.77) as well as with the adjusted approach, which accounted for both double counting (i.e., two studies shared one comparison) and variance inflation due to studies with moderate and high risk of bias (HR: 0.56; 95% CI: 0.39, 0.82). In the absence of clinical trials with head‐to‐head comparison of these treatment regimens, these results could help inform the selection of optimal first‐line treatment for TIE NDMM patients.

## 1 Introduction

Multiple myeloma (MM) makes up 1.8% of all cancer diagnosis in the US, with an incidence rate of 7.0 per 100,000 individuals [1]. MM is a multifactorial disease with risk factors such as male sex, increased age, prior family history, pernicious anemia, genetic factors, and occupational factors [2]. Common complications associated with MM include various hematological concerns (e.g., anemia), bone‐related conditions (e.g., spinal cord compressions and pathological fractures), neurological symptoms, infections, and renal insufficiency [3].

Treatment of MM varies based on whether a patient is eligible for stem cell transplantation (SCT) or not [4, 5]. Transplant eligibility is determined by age, performance status, and comorbidities as elderly, more frail individuals are more prone to treatment‐related toxicity making them ineligible [6]. Before the introduction of daratumumab, a CD38 targeted monoclonal antibody, bortezomib in combination with lenalidomide, and dexamethasone (VRd), bortezomib with melphalan and prednisone (VMP), or lenalidomide with dexamethasone (Rd) were commonly prescribed in transplant‐ineligible (TIE) newly diagnosed MM (NDMM) [5]. In 2019, daratumumab in combination with lenalidomide, and dexamethasone (DRd) was approved by the Food and Drug Administration (FDA) in TIE NDMM patients based on results from the MAIA trial, an international randomized phase III, active‐controlled trial. In the MAIA trial, patients treated with DRd demonstrated significantly lowered risk of disease progression or death versus Rd [7].

Currently, DRd and VRd are the only preferred triplet therapy options recommended by the NCCN Clinical Practice Guidelines in Oncology (NCCN Guidelines) for the primary treatment of TIE patients with NDMM [8]. It is unclear whether DRd or VRd should be prioritized as primary treatment for TIE NDMM due to differences in their efficacy and safety, as well as the absence of head‐to‐head trials comparing DRd and VRd directly. Despite the insights provided by several network meta‐analyses (NMAs) on existing therapies for this patient population [9, 10, 11, 12, 13], they come with limitations including not sufficiently accounting for heterogeneity among trials, lack of long‐term follow‐up data for some of the trials, inclusion of treatments not used in routine clinical practice in the US, and use of aggregate level data from the pivotal trials. Additionally, the published NMAs only focused on randomized controlled trials (RCTs) while excluding real‐world evidence (RWE) studies. This approach may overlook insights from routine clinical practice, where factors such as patient diversity, comorbidities, and treatment adherence can significantly impact outcomes. Furthermore, the rapidly evolving landscape of clinical data in TIE NDMM patients highlights the need for updated evidence. RWE studies focus on real‐world clinical practices and address the timeliness concern highlighted in existing NMAs. Combining findings across both RCTs and RWE studies that directly and indirectly compare effectiveness of treatments could provide a comprehensive understanding of treatment outcomes, which could guide clinical decision‐making regarding treatment selection of TIE NDMM patients.

The primary objective of this study was to systematically identify evidence on the clinical effectiveness of DRd and VRd as first‐line treatments for patients with TIE NDMM across a wide evidence base, including RCTs, RWE studies, and indirect treatment comparisons (ITCs) and to conduct a comprehensive meta‐analysis (MA) examining the efficacy of DRd versus VRd.

## 2 Methods

This systematic literature review (SLR) was performed in accordance with the Cochrane Handbook for Systematic Reviews of Interventions [14] and reported consistent with the Preferred Reporting Items for Systematic Reviews and Meta‐Analyses (PRISMA) statement [15, 16]. The study protocol was registered on PROSPERO (registration number CRD42023435119).

### 2.1 Search Strategy

The search strategy (Supporting Information Table S1) was developed based on the pre‐defined PICOS criteria by a medical information specialist and peer‐reviewed independently by another senior medical information specialist before execution using the Peer Review of Electronic Search Strategies (PRESS) checklist [17].

Using the Ovid search interface, Ovid MEDLINE (including Epub Ahead of Print and In‐Process & Other Non‐Indexed Citations), Embase, the Cochrane Central Register for Controlled Trials, and the Cochrane Database of Systematic Reviews were searched. Searches were restricted to English‐language articles published between January 1, 2019, and June 14, 2023, and conference abstracts published between January 1, 2018, and June, 2023. Detailed searches of conference websites were also performed to identify abstracts from key conferences held during the same period. The following conferences were searched: International Myeloma Society, American Society of Hematology (ASH), European Hematology Association (EHA), and American Society of Clinical Oncology (ASCO). A hand search of bibliographies of relevant recent SLRs, MAs, and NMAs identified via the original database search was conducted to ensure the inclusion of all relevant studies. ClinicalTrials.gov was searched to identify studies investigating DRd versus VRd. Additionally, a post‐SLR literature scan was performed to identify any new studies or recent publications related to included studies, and relevant publications were included. The date restriction was chosen to align with the FDA and European Medicines Agency (EMA) approvals of DRd for TIE NDMM in 2019 [18, 19].

### 2.2 Study Eligibility Criteria

Full‐text publications and conference abstracts of English‐language studies (including clinical trials and appropriately adjusted, non‐randomized comparative studies) were eligible if they enrolled TIE NDMM patients and compared DRd and VRd as first line of treatment. Outcomes of interest included overall survival (OS), progression‐free survival (PFS), overall response rate (ORR), complete response (CR), and partial response (PR), among others (Table 1).

**TABLE 1:** PICOS criteria for inclusion and exclusion of studies.

Item	Inclusion criteria	Exclusion criteria
Population	Patients aged ≥ 18 years NDMM Ineligible for SCT	Patients aged < 18 years RRMM Eligible for SCT
Intervention	DRd	Those not listed as inclusion
Comparator	VRd VRd lite	Those not listed as inclusion
Outcomes	Survival Endpoints PFS OS Response endpoints Overall response rate Complete response or better (stringent complete response, complete response) Very good partial response or better, very good partial response Time to progression Time to next treatment Negative status for minimal residual disease Treatment discontinuations (proportions)	Those not listed as inclusion
Study design	RCTs Adequately adjusted non‐randomized comparative studies (retrospective/prospective cohort study, [including but not limited to chart review, EMR, claims database], cross‐sectional, case‐control, registries), non‐interventional studies, MAs, ECAs and ITCs.	Animal studies, in vitro studies, case reports, case series, expert opinion articles, commentaries, letters, protocols, single‐arm studies SLRs^a^
Date range	2019‐present (2018‐present for abstracts only)	Literature pre‐2018
Region	Any region	None
Language	English^b^	All other languages^b^

Abbreviations: AE = adverse event, DRd = daratumumab + lenalidomide + dexamethasone, ITC = indirect treatment comparisons; MA = meta‐analysis; MM = multiple myeloma, NDMM = newly diagnosed multiple myeloma, OS = overall survival, PFS = progression‐free survival, RCT = randomized controlled trial, RRMM = relapsed/refractory multiple myeloma; SCT = Stem‐cell transplantation, SLR = systematic literature review; VRd = bortezomib + lenalidomide + dexamethasone.

^a^ On‐topic SLRs were tracked during study screening, and bibliographies of select recent SLRs were reviewed for relevant studies.

^b^ Language filters were not applied at the search stage. Non‐English articles were excluded during screening.

### 2.3 Study Selection

Studies were screened based on PICOS criteria established a priori (Table 1). Two independent reviewers (MS and NN) screened titles and abstracts and full texts for eligibility. Conflicts were resolved by consensus and/or a third reviewer (AOR). Hand searches and study selection of all gray literature sources were conducted by a single reviewer and verified by a second reviewer.

### 2.4 Data Extraction

Data were extracted by two independent reviewers. A standardized extraction form was used for capturing the data. The following data were extracted: publication characteristics (e.g., year, study sponsor, objective); study setting (e.g., countries, centers/hospitals); study methods (e.g., design, follow‐up length, patient enrollment criteria); study treatments (e.g., interventions, dosing regimens, treatment duration); study participants characteristics (e.g., age, sex, Eastern Cooperative Oncology Group Performance Status (ECOG PS), International Staging System (ISS) stage, standard/high‐risk cytogenetics); study findings (e.g., OS, PFS, ORR).

### 2.5 Risk of Bias

The risk of bias for each study was assessed using checklists recommended by the National Institute for Health and Care Excellence (NICE) for non‐randomized and non‐controlled studies [20]. Risk of bias assessments were conducted by a single reviewer, and a second reviewer validated the findings.

Since the NICE checklist does not provide a method to assign an overall risk of bias score, guidance from the Risk of Bias in Non‐randomized Studies of Interventions (ROBINS‐I) checklist was followed [21]. According to this guidance, a study was deemed to be at a “low risk” of bias if the study was comparable to a high‐quality RCT across all domains. A study was deemed to be at an “unclear/moderate risk” of bias if the study provided sound evidence for its study design but could not be considered comparable to a high‐quality RCT in at least one domain. A study was deemed to be at a “high risk” of bias if the study had significant issues in at least one domain which bias outcomes in favor of one of the treatments.

### 2.6 Statistical Analysis

Relative effect sizes were pooled to report the overall relative treatment effect of DRd versus VRd on PFS, the only outcome reported across the included studies. Because two of the included studies used anchored ITCs for the comparison of DRd versus VRd and shared one comparison (i.e., MAIA RCT), guidance from the Cochrane collaboration was followed to account for the correlation when pooling these studies [22]. Since the repeated patients were judged to be identical or near identical between comparisons, the standard error from the version with longer follow‐up was inflated to divide patients between the ITCs to avoid the issue of double counting. In addition, results of the risk of bias assessments were used to inflate the variance of higher risk of bias studies relative to lower risk of bias studies as suggested by Efthimiou et al. (2017) [23]. Low risk of bias studies had no variance inflation, but unclear/moderate risk of bias studies had their variance inflated by 25% (i.e., $\frac{s_{\text{moderate}}^{2}}{0.75}$), and high risk of bias studies had variance inflated by 50% (i.e., $\frac{s_{\text{moderate}}^{2}}{0.5}$). Finally, two fixed‐effect MAs were performed to combine the estimates using inverse variance weights: (a) the naïve approach, with no adjustments for double counting and variance inflation, and (b) the adjusted approach, accounting for both double counting and variance inflation. Pooled hazard ratios (HR) and 95% confidence intervals (CI) were reported. The analyses were conducted using the *meta* package in R [24].

## 3 Results

**FIGURE 1:**
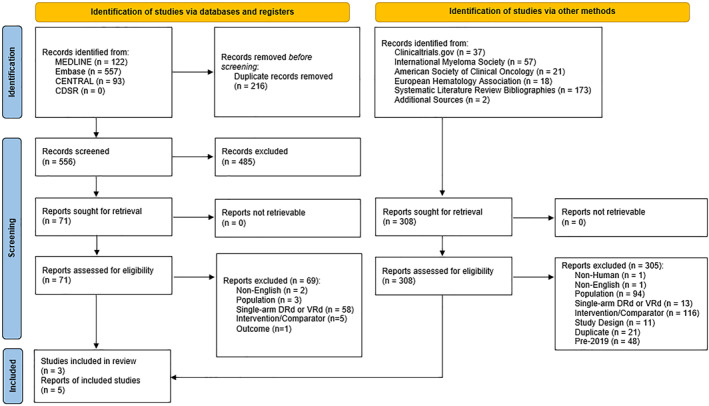
PRISMA flow diagram. CDSR = Cochrane Database of Systematic Reviews; DRd = daratumumab + lenalidomide + dexamethasone; PRISMA = Preferred Reporting Items for Systematic Reviews and Meta‐Analyses; VRd = bortezomib + lenalidomide + dexamethasone.

A total of 1080 records were identified, 772 through the database search and 308 through hand searches. From the records identified through database search, 216 duplicates were removed. The resulting 556 records were screened at the title and abstract stage and 485 were further excluded. The remaining 71 records were retrieved and assessed for eligibility. A total of 69 records were excluded for the reasons shown in the PRISMA flow diagram (Figure 1).

All 308 records that were identified through hand‐searching of clinical trials registries (ClinicalTrials.gov), selected conferences, SLR bibliographies, and other sources were retrieved and assessed for eligibility. Of these, 305 were excluded for various reasons (Figure 1). In total, five records reporting on three unique studies were included in this review.

### 3.1 Risk of Bias

Of the five included records, a total of three unique studies were assessed for risk of bias (Supporting Information Table S2). Key limitations of the included studies were related to confounding and missing data. All three studies were limited in the selection of confounders they reported; in particular, the suitability of the TIE definition used in PEGASUS could not be established [25]. Although efforts were made across all studies to address issues related to missing data, their study designs did not meet the criteria to be classified as high‐quality RCTs. There was no evidence of selective reporting bias across included studies. Overall, it was deemed that PEGASUS had a high risk of bias, whereas TAURUS and MAIA versus SWOG S0777 had a moderate risk of bias.

### 3.2 Study Characteristics

One study was a retrospective chart review (TAURUS) [26], and two studies were ITCs (PEGASUS and MAIA vs. SWOG S0777) [25, 27]. The two ITCs had an overlapping data source (i.e., the MAIA RCT) [7]. Studies varied with respect to sample size (i.e., 178 to 2075 patients) [25, 26], start of patient enrollment period (i.e., 2008–2019) [26, 27], and maximum follow‐up (24–80 months) [26, 27] (Table 2).

**TABLE 2:** Study characteristics.

Study characteristics	TAURUS [26]	MAIA versus SWOG S0777 [27]		PEGASUS [25]	
		MAIA	SWOG S0777	MAIA	Flatiron I
*N* patients	178	925^a^		2075^a^	
Study design	Retrospective chart review	Anchored ITC		Anchored ITC	
Data sources	9 large academic and community‐based cancer centers in the US	IPD from MAIA RCT (NCT02252172)	IPD from SWOG S0777 RCT (NCT00644228)	IPD from MAIA RCT (NCT02252172)	IPD from US flatiron health I—derived deidentified database
Country/region	USA	14 countries across North America, Europe, the Middle East, and the Asia–Pacific region	Puerto Rico, Saudi Arabia, USA	14 countries across North America, Europe, the Middle East, and the Asia–Pacific region	USA
Enrollment	January 2019 to September 2021	March 2015 to January 2017	April 2008 to February 2012	March 2015 to January 2017	January 1, 2011 to April 30, 2019
Maximum follow‐up (months)	24	80		48.5	
Treatments	DRd versus VRd	DRd versus Rd	VRd versus Rd	DRd versus Rd	VRd versus Vd versus Rd

Abbreviations: ECA = external control arm; HSCT = hematopoietic stem cell transplantation; I = electronic health records; IPD = individual patient data; ITC = indirect treatment comparison; NR = not reported; RCT = randomized controlled trial; USA = United States of America.

^a^ Calculated value.

### 3.3 Patient Characteristics

The following patient and disease characteristics were reported in the three included studies: age, sex, ISS stage, ECOG PS, and cytogenetic risk. Other patient and disease characteristics such as race and mean time from MM diagnosis to treatment were more sparsely reported. Only one study reported data on hemoglobin, estimated glomerular filtration rate, and lactate dehydrogenase, and none of the studies reported data on type of MM (Table 3) [27].

**TABLE 3:** Summary of baseline characteristics.

Patient characteristic	TAURUS [26]		MAIA versus SWOG S0777 [27]		PEGASUS [25]	
	DRd (*n* = 91)	VRd (*n* = 87)	DRd versus Rd (*n* = 727)	VRd versus Rd (*n* = 198)	DRd (*n* = 358)	VRd (*n* = 570)
Age in years, mean (SD)	76.2 (5.8)	75.9 (6.1)	72.7 (4.8)	72.7 (5.2)	74.2 (5.1)	74.3 (5.3)
Female (%)	51.1	52	41	39	48.9	48.5
Race (%)	White: 53.1 Black: 14.2 Other: 32.7	White: 53 Black: 14.8 Other: 32.2	NR	NR	Black: 2.5 Other: 97.5	Black: 3.4 Other: 96.6
ISS stage (%)	I: 24.5 II: 29.9 III: 17.7 Unknown: 27.9	I: 27 II: 24.4 III: 17.2 Unknown: 31.5	I: 20 II: 45 III: 35	I: 20 II: 45 III: 35	I: 27.1 II: 45.5 III: 27.4	I: 27.7 II: 43.5 III: 28.8
ECOG PS (%)	0: 32 1‐2: 57.7 3‐4: 2.7 Unknown: 7.7	0: 31.7 1‐2: 58 3‐4: 3.7 Unknown: 6.6	0: 42 1: 50 2: 9	0: 40 1: 51 2: 9	0: 35.2 1: 48.6 2: 16.2	0: 35.2 1: 49.1 2: 15.7
High‐risk cytogenetics (%)	14.4	17.2	19	17	15.1	14.1
Mean time from MM diagnosis to treatment (months)	3.3	3.3	NR	NR	1.4	1.5
Hemoglobin < 10 g/dL (%)	NR	NR	31	32	NR	NR
eGFR < 60 mL/min/1.73 m^2^ (%)	NR	NR	47	47	NR	NR
LDH ≥ 190 U/L (%)	NR	NR	39	39	NR	NR
Frailty score ≥ 2^a^	60.3	60.6	NR	NR	NR	NR

Abbreviations: DRd = daratumumab + lenalidomide + dexamethasone; ECOG PS = Eastern Cooperative Oncology Group performance status; eGFR = estimated glomerular filtration rate; LDH = lactate dehydrogenase MM = multiple myeloma NR = not reported; U/L = units per liter; VRd = bortezomib + lenalidomide + dexamethasone.

^a^ Calculation of frailty is based on three components: Age (≤ 75 years = 0 point, 76–80 years = 1 point, > 80 years = 2 points), CCI (≤ 1 = 0 point, > 1 = 1 point), and ECOG performance status (0 = 0 point, 1 = 1 point, ≥ 2 = 2 points).

The mean age across included studies ranged from 72.7 to 76.2 years [26, 27]. The percentage of females enrolled in the studies ranged from 39% to 52% [26, 27]. The proportion of patients with advanced disease stage (ISS III) ranged from 17.2% to 35% [26, 27]. Two studies exclusively enrolled patients with ECOG PS ≤ 2 [25, 27] and one study included a percentage of patients with ECOG PS 3‐4 [26]. The proportion of patients with high‐risk cytogenetics ranged from 14.1% to 19% (Table 3) [25, 27].

### 3.4 Study Outcome

Progression‐free survival (PFS), defined as time from index date or first‐line therapy/randomization until disease progression or death, was the only outcome of interest reported across the included studies. All three studies reported adjusted hazard ratios for PFS comparing DRd versus VRd. Additionally, one study reported PFS rates for patients who received VRd and DRd at 6 and 12 months [26]. In each study, TIE NDMM patients treated with DRd had a significantly lower risk of progression or death compared to those treated with VRd (Table 4).

**TABLE 4:** Summary of study outcomes.

Study name	*N*	PFS of DRd versus VRd		
		HR	95% CI	*p*‐value
TAURUS [26]	DRd: 91 VRd: 87	0.35	0.17, 0.73	0.005
MAIA versus SWOG S0777 [27]	DRd: 727 VRd: 198	0.60	0.39, 0.90	0.02
PEGASUS [25]	DRd: 358 VRd: 570	0.68	0.48, 0.98	0.04

Abbreviations: CI = confidence interval; DRd = daratumumab + lenalidomide + dexamethasone; HR = hazard ratio; PFS = progression‐free survival; VRd = bortezomib + lenalidomide + dexamethasone.

### 3.5 Meta‐Analysis

The three studies identified via the SLR were included in the MA for PFS. The fixed‐effects MA using the naïve approach showed a 40% lower risk of disease progression or death with DRd compared to VRd (HR: 0.60; 95% CI: 0.46, 0.77; *I* ^*2*^ = 22%).

**FIGURE 2:**
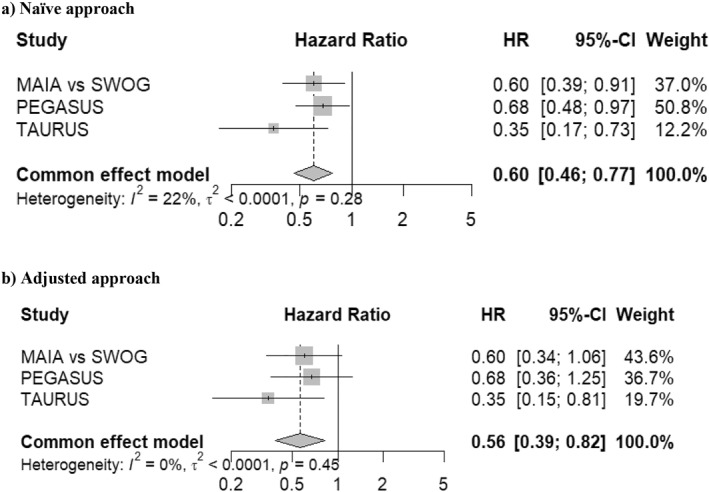
Forest plots from fixed‐effects meta‐analysis of progression‐free survival (PFS) comparing DRd vs. VRd (a) Naïve approach without adjustment for double counting and inflation factor from risk of bias assessment and (b) adjusted approach accounting for double counting and inflation factor from risk of bias assessment.

However, to account for the risk of bias assessment, which categorized PEGASUS as having a high risk of bias and TAURUS and MAIA versus SWOG S0777 a moderate risk of bias, an adjusted analysis was performed. The variance of the effect estimates were inflated by 50% for PEGASUS and 25% for TAURUS and MAIA versus SWOG S0777. Furthermore, the adjusted analysis accounted for the potential double counting in the pooled results originating from the inclusion of the MAIA trial in both PEGASUS and anchored the ITC versus SWOG S0777, with an adjustment for variance of the DRd comparison (multiplied by 2). The pooled results for both the naïve and unadjusted analysis were similar, with the adjusted results showing a 44% lower risk of disease progression or death (HR: 0.56; 95% CI: 0.39, 0.82; *I* ^*2*^ = 0%) (Figure 2).

## 4 Discussion

This SLR and MA was performed to identify and aggregate clinical evidence specifically comparing DRd versus VRd as first‐line treatment for TIE patients with NDMM incorporating a wide range of evidence, including ITCs and RWE studies. A total of five records, representing three unique studies, were identified. In terms of study quality, limitations were most noted for the domains of study confounding and missing data. Studies varied in terms of study design characteristics and key patient baseline characteristics, such as the ECOG PS and ISS. Additionally, data on important patient baseline characteristics (e.g., hemoglobin, estimated glomerular filtration rate, and lactate dehydrogenase) were only reported by one study [27]. In each study, TIE NDMM patients treated with DRd had a significantly lower risk of progression or death compared to those treated with VRd.

Despite methodological and clinical heterogeneity, a detailed and standard approach was adopted to address issues identified during the qualitative review of the evidence base which included double counting and differences in risk of bias. Results from the fixed effects MA showed that patients treated with DRd as first‐line treatment had approximately 40% lower risk of progression or death compared to those treated with VRd in the naïve analysis and a 44% lower risk in the adjusted analysis.

The dearth of studies directly comparing the effectiveness of DRd vs. VRd underscores the need for a comprehensive update of the current SLR to identify and incorporate new evidence that may have emerged since the completion of this review, given the rapidly evolving landscape of clinical data within this disease area. This updated synthesis should continue to integrate RWE studies to provide a comprehensive understanding of the relative merits of DRd vs. VRd in the contemporary clinical context. While RCTs remain the gold standard for evaluating efficacy and safety under controlled conditions, real‐world studies provide valuable complementary information by assessing the effectiveness of treatments as used by patients and clinicians. Moreover, the limitations of real‐world studies continue to be better understood and improved methodologies are increasingly addressing these challenges.

The findings of this SLR and MA contribute valuable insights into the comparative effectiveness of DRd vs. VRd as first‐line treatments for TIE NDMM. These results could have clinical implications for the management of TIE NDMM, as clinicians may consider these findings when making treatment decisions for patients with this condition. These insights should be further complemented by pragmatic considerations such as patient preferences regarding dosing schedules and drug tolerability, to ensure a holistic approach to treatment decision making.

Limitations of this review include the potential risk of increased bias due to the inclusion of non‐randomized observational studies. Additionally, the inclusion of evidence from various study designs such as RCTs, RWE, and ITCs may increase the degree of heterogeneity. However, this concern was mitigated by restricting the analysis to studies aligned with a common underlying target trial. Another limitation was the restriction to English‐language articles only. However, given that most of the key studies identified were published in English journals, it is likely that this was a minor limitation. Despite these limitations, this review exhibits notable strengths. A key strength was its adherence to best practices for conducting and reporting systematic reviews. Notably, all database searches were performed and peer‐reviewed by an experienced medical information specialist. As per the PRISMA statement, the current review reports detailed search strategies, PICOS, a PRISMA flow diagram, and risk of bias assessments using appropriate tools. Additionally, this review included multiple study designs, such as ITCs and a retrospective chart review. Rigorous methodological steps were implemented to ensure the robustness of the analysis. Specific attention was given to resolving discrepancies in study characteristics to enhance internal validity and promote external comparability across the selected studies.

## 5 Conclusion

The results from the individual studies identified in the SLR and the aggregate estimate from the MA showed that DRd is associated with a lower risk of disease progression or death compared to VRd as first‐line treatment for TIE patients with NDMM. This MA used a comprehensive approach, incorporating evidence from both RCTs and RWE. Findings from this study could help inform the selection of first‐line treatment for TIE patients with NDMM, especially in the absence of head‐to‐head clinical trials. Additionally, these findings highlight the potential value of including RWE to complement RCTs when estimating treatment effectiveness, as these estimates are more reflective of real‐world practice.

## Author Contributions

All authors made substantive intellectual contributions to this study to qualify as authors. L.N.G., R.M., A.Z.F., M.Sh., A.O.R., N.B., B.‐N.N., M.Sp., R.M.T., T.D., S.G., N.G.‐W., S.K., and A.J.P. participated in study design through drafting or approval of the protocol. B.‐N.N and M.Sp. contributed to the literature search. B.‐N.N. and M.Sp. worked on data collection. N.B. and T.D. analyzed and interpreted the data. M.Sh., A.O.R., and M.Sp. wrote the manuscript draft. R.M. and S.G. assisted with manuscript revisions. All authors reviewed and approved the final version of the manuscript.

## Ethics Statement

The authors have nothing to report.

## Conflicts of Interest

R.M., A.Z.F., S.G., N.G.‐W., and S.K. are employed by Janssen Scientific Affairs, L.L.C., and may own Johnson & Johnson stock/stock options. M.Sh., A.O.R., N.B., B.‐N.N., M.Sp., R.M.T., and T.D. are employed by EVERSANA, Canada, which was contracted by Janssen Scientific Affairs, L.L.C. to work on this study.

### Peer Review

The peer review history for this article is available at https://www.webofscience.com/api/gateway/wos/peer-review/10.1002/hon.70061.

## Supporting information

Supporting Information S1

## Data Availability

This was a secondary data analysis study and all data for the analyses are reported.
