# Supplementary material for: Daratumumab, Lenalidomide, and Dexamethasone Versus Bortezomib, Lenalidomide, and Dexamethasone in Transplant‐Ineligible Newly Diagnosed Multiple Myeloma: A Systematic Literature Review and Meta‐Analysis
Source: Hematol Oncol. 2025 Apr 10;43(3):e70061. doi: 10.1002/hon.70061 (PMC11984075; doi:10.1002/hon.70061)
Supplement: Supplementary file 1 — Supporting Information S1 [file HON-43-e70061-s001.docx]

# Supplementary Tables

Supplemental Table 1: Search Strategy

| **#** | **Searches** | **Results** |
| --- | --- | --- |
| 1 | exp Multiple Myeloma/ or (((myelom$ or leuk?em$) adj4 (multiple or multiplex or plasma-cell? or plasmacell$ or plasmacyt$)) or "myeloma-multiple?" or kahler$ or myelomatos#s or (MM and (myeloma? or leuk?emi$))).ti,ab,kw,kf. [MULTIPLE MYELOMA TERMS] | 185576 |
| 2 | exp Hematopoietic Stem Cell Transplantation/ or (((h?ematopoietic or h?emato-poietic) adj3 stem-cell? adj3 (transplant$ or therap$ or transfer$)) or ((HCT? or HSC? or HSCT? or allo-HSC$ or alloHSC$ or auto-HSC$ or autoHSC$) and (transplant$ or therap$ or transfer$ or allogeneic$ or allogenic$ or allo-geneic$ or allo-genic$ or peripheral$ or autologous)) or (transplant$ adj2 (ineligibl$ or "not eligible" or non-eligible or "not qualified" or "not suitable" or "not appropriate" or "not fit" or TIE or NTE)) or (non-transplant$ adj2 (MM or multiple myelom$ or patient? or candidate?))).ti,ab,kw,kf. [HSCT or TRANSPLANT-INELIGIBLE TERMS] | 227304 |
| 3 | 1 and 2 [MM & HSCT TERMS] | 14665 |
| 4 | (daratumumab$2 or dalinvi$2 or darasarex$2 or darzalex$2 or hlx-15 or hlx15 or humax-CD38 or humax-CD-38 or jnj-54767414 or jnj54767414 or 945721-28-8 or 4Z63YK6E0E).ti,ab,kw,kf,ot,hw,rn,nm. [DARATUMUMAB TERMS] | 8480 |
| 5 | Bortezomib/ or (bortezomib$ or brotezamide$2 or milatib$2 or mylosome$2 or velcad$2 or radiciol$2 or "peptide boronate" or "proscript boronic acid" or pyz-phe-boroleu or bxcl-101 or bxcl101 or hsb-407 or hsb-408 or hsb407 or hsb408 or jnj-26866138 or jnj26866138 or 324b697 or 3mg0 or a18332 or ab01273951-03 or ab01273951-01 or ab01273951-02 or akos015909706 or am81235 or as-15721 or b5741 or bb164258 or bdbm50069989 or brd-k88510285-001-02-0 or c19h25bn4o4 or ccg-268449 or chebi-52717 or chembl325041 or cs-1039 or d03150 or db00188 or dpba or dtxsid3040980 or en300-657180 or gtpl6391 or hb4036 or hy-10227 or mfcd09056737 or mln-341 or mls004774142 or ncgc00168751-01 or ncgc00181022-01 or ncgc00242506-01 or ncgc00242506-02 or ncgc00242506-06 or ncgc00242506-07 or nci60-029010 or nsc756655 or nsc-756655 or q419319 or s1013 or schembl192129 or smr003500787 or sr-01000939863 or sr-01000939863-2 or sw208077-3 or tox21-112630 or tox21-112672 or tox21-112672-1 or z2213886907 or zinc169746649 or nsc-681239 or nsc681239 or mg-341 or mg341 or lpd341 or lpd-341 or ldp-341 or ldp341 or hsdb-7666 or mln-341 or mln341 or ps-341 or ps341 or 341-ps or 179324-69-7 or 197730-97-5 or 444576-08-3 or 1610526-91-4 or 69G8BD63PP).ti,ab,kw,kf,ot,hw,rn,nm. [BORTEZOMIB TERMS] | 54560 |
| 6 | Lenalidomide/ or (lenalidomid$ or revimid$2 or revlimid$2 or ladevina$2 or 732L726 or ab01273975-03 or ab01273975-01 or ab01273975-02 or ac-914 or akos005146276 or akos005174869 or albb-015321 or am20050439 or bcp01390 or bcp9000847 or bcpp000186 or bdbm65454 or bl164614 or bp-27972 or c13h13n3o3 or ccg-264781 or chebi-63791 or chembl848 or cs-0125 or d04687 or db00480 or dtxsid8046664 or ec-000-2340 or en300-118706 or f0p408n6v4 or ft-0659651 or ft-0670758 or ft-0670759 or gtpl7331 or hms3654g07 or hms3674c05 or hy-a0003 or imid-5013 or ks-1207 or mfcd07772307 or mls003899194 or ncgc00167491-01 or ncgc00167491-02 or ncgc00167491-03 or ncgc00167491-04 or nsc747972 or nsc-747972 or q-101410 or q425681 or s1029 or sb66166 or schembl1980410 or schembl32978 or smr002529986 or sr-01000883999 or sr-01000883999-1 or stk639603 or sw218084-2 or sy047646 or syp-1512 or syp1512 or tox21-112492 or tox21-112492-1 or z1515385074 or hsdb-8220 or hsdb8220 or cc-5013 or cc5013 or cdc-501 or cdc-5013 or cdc501 or cdc5013 or enmd-0997 or enmd0997 or imid-3 or imid3 or 191732-72-6 or 1243329-97-6 or 202271-91-8 or 874946-00-6 or 443912-14-9 or F0P408N6V4).ti,ab,kw,kf,ot,hw,rn,nm. [LENALIDOMIDE TERMS] | 36128 |
| 7 | Dexamethasone/ or (dexamethason$ or adrecort$2 or 064d136 or 1p93 or ab00918428-05 or ab00918428-08 or ab00918428-09 or ab00918428-10 or ac-11056 or adrenocot$2 or aeroseb-d$2 or aeroseb-dex$2 or aflucoson$2 or aflucosone$2 or ai3-50934 or akos005259009 or akos015895509 or alfalyl$2 or amy28815 or anaflogistico$2 or aphtasolon$2 or aphthasolone$2 or apo-dexamethasone$2 or arcodexan$2 or arcodexane$2 or artrosone$2 or auxiron$2 or azium$2 or baycadron$2 or bdbm18207 or bidd-er0494 or bidd-pxr0060 or bidexol$2 or bisu-ds$2 or brd-k38775274-001-02-3 or brd-k38775274-001-06-4 or bspbio-000995 or calonat$2 or ccg-264887 or ccris-7067 or cebedex$2 or cetadexon$2 or chebi-41879 or chembl384467 or colofoam$2 or corsona$2 or corsone$2 or cortastat$2 or cortidex$2 or cortidexason$2 or cortidrona$2 or cortidrone$2 or cortisumman$2 or cs-0626118 or cs-1505 or d00292 or dacortina-fuerte$2 or dacortine-fuerte$2 or dalalone$2 or danasone$2 or db01234 or decacort$2 or decacortin$2 or decadeltosona$2 or decadeltosone$2 or decaderm$2 or decadion$2 or decadran$2 or decadron$2 or decadronal$2 or decadrone$2 or decaesadril$2 or decagel$2 or decaject$2 or decalix$2 or decameth$2 or decamethasone$2 or decasone$2 or decaspray$2 or decasterolone$2 or decdan$2 or decilone$2 or decofluor$2 or dectancyl$2 or dekacort$2 or delladec$2 or deltafluoren$2 or deltafluorene$2 or dergramin$2 or deronil$2 or desacort$2 or desacortone$2 or desadrene$2 or desalark$2 or desameton$2 or desametone$2 or deseronil$2 or desigdron$2 or de-sone-la$2 or dexa-cortisyl$2 or dexa-dabrosan$2 or dexa-korti$2 or dexa-mamallet$2 or dexa-scherosan$2 or dexa-scherozon$2 or dexa-scherozone$2 or dexacen-4$2 or dexachel$2 or dexacidin$2 or dexacort$2 or dexacortal$2 or dexacorten$2 or dexa-cortidelt$2 or dexacortin$2 or dexacortisyl$2 or dexa-cortisyl$2 or dexadabroson$2 or dexadecadrol$2 or dexadeltone$2 or dexadrol$2 or dexafarma$2 or dexagel$2 or dexagen$2 or dexahelvacort$2 or dexakorti$2 or dexalien$2 or dexalocal$2 or dexalona$2 or dexame$2 or dexamecortin$2 or dexameson$2 or dexamesone$2 or dexametason$2 or dexametasone$2 or dexameth$2 or dexamethason$2 or dexamethazon$2 or dexamethazone$2 or dexamethonium$2 or dexamonozon$2 or dexan$2 or dexane$2 or dexano$2 or dexa-p$2 or dexapolcort$2 or dexapos$2 or dexapot$2 or dexaprol$2 or dexascheroson$2 or dexa-scheroson$2 or dexascherozon$2 or dexa-scherozon$2 or dexascherozone$2 or dexa-sine$2 or dexasite$2 or dexason$2 or dexasone$2 or dex-ide$2 or dexinolon$2 or dexinoral$2 or dexionil$2 or dexmethsone$2 or dexona$2 or dexone$2 or dexone$2 or dexpak$2 or dextelan$2 or dextenza$2 or dextrasone$2 or dexycu$2 or dezone$2 or dibasona$2 or dinormon$2 or diodex$2 or doxamethasone$2 or dtxsid3020384 or dxms$2 or einecs-200-003-9 or en300-52607 or esacortene$2 or ex-s1 or exadion$2 or exadione$2 or firmalone$2 or fluormethyl-prednisolone$2 or fluormethylprednisolone$2 or fluormethyl-prednisolon$2 or fluormethylprednisolon$2 or fluoromethylprednisolone$2 or fluoromethyl-prednisolone$2 or fluormone$2 or fluorocort$2 or fluorodelta$2 or fortecortin$2 or gammacorten$2 or gammacortene$2 or grosodexon$2 or grosodexone$2 or gtpl2768 or gtpl3447 or hb2521 or hemady$2 or hexadecadiol$2 or hexadecadrol$2 or hexadiol$2 or hexadrol$2 or hl-dex or hms1792a17 or hms1990a17 or hms2089n13 or hms2235f08 or hms3039l11 or hms3259n11 or hms3403a17 or hsdb 3053 or hy-14648 or hy-14648g or ibi-10090 or isnacort$2 or isoptodex$2 or isopto-dex$2 or isoptomaxidex$2 or isopto-maxidex$2 or isv-305 or isv305 or ks-1451 or ks1451 or lokalison-f$2 or loverine$2 or luxazone$2 or marvidione$2 or maxidex$2 or maxitrol$2 or mediamethasone$2 or megacortin$2 or mephameson$2 or mephamesone$2 or metasolon$2 or metasolone$2 or methazon-ion$2 or methazone-ion$2 or methazonion$2 or methazonione$2 or methylfluorprednisolone$2 or methylfluor-prednisolone$2 or metisone-lafi$2 or mexasone$2 or mexidex$2 or mfcd00064136 or millicorten$2 or millicortenol$2 or mk-125 or mk125 or mls001055412 or mls001332507 or mls001332508 or molmap-000018 or mymethasone$2 or nc00645 or ncgc00091019-01 or ncgc00091019-02 or ncgc00091019-03 or ncgc00091019-04 or ncgc00091019-05 or ncgc00091019-06 or ncgc00091019-07 or ncgc00091019-08 or ncgc00091019-23 or ncgc00257676-01 or nci60-003067 or neoforderx$2 or neofordex$2 or nisomethasona$2 or novocort$2 or nsc-34521 or nsc34521 or ocu-trol$2 or oftan-dexa$2 or opticorten$2 or opticortinol$2 or oradexan$2 or oradexon$2 or oradexone$2 or orgadrone$2 or osurdex$2 or oto-104 or oto104 or ozurdex$2 or pet-derm-iii$2 or phl-dexamethasone$2 or pidexon$2 or policort$2 or posurdex$2 or predni-f-tablinen$2 or predni-f$2 or prednisolon-f$2 or prednisolone-f$2 or prodex$2 or prodexona$2 or prodexone$2 or q-200939 or q422252 or s1322 or sanamethasone$2 or santenson$2 or santeson$2 or sawasone$2 or schembl3774 or sgcut00126 or sk-0503 or sk0503 or smp1-000092 or smr000857119 or smr001227192 or solurex$2 or spectrum5-002019 or spoloven$2 or spt-2101 or spt2101 or sterasone$2 or sunia-sol-d$2 or superprednol$2 or thilodexine$2 or to-000038 or tox21-200122 or triamcimetil$2 or turbinaire$2 or vexamet$2 or visumetazone$2 or visumethazone$2 or zema-pak$2 or zinc3875332 or 23495-06-9 or 1950-02-02 or 1050677-47-8 or 137098-19-2 or 8054-59-9 or 906362-70-7 or 906422-84-2 or 50-02-2 or Z756391748 or 7S5I7G3JQL).ti,ab,kw,kf,ot,hw,rn,nm. [DEXAMETHASONE TERMS] | 303472 |
| 8 | (4 and 6 and 7) or DRd.ti,ab,kw,kf. [DRd TERMS] | 4613 |
| 9 | (5 and 6 and 7) or VRd.ti,ab,kw,kf. [VRd TERMS] | 11066 |
| 10 | 3 and 8 [MM & DRd TERMS] | 439 |
| 11 | 3 and 9 [MM & DRd TERMS] | 1317 |
| 12 | 10 or 11 [MM & DRd or VRd TERMS] | 1413 |
| 13 | (randomized controlled trial or controlled clinical trial).pt. or (randomized or placebo or randomly or trial or groups).ti,ab. or drug therapy.fs. [RCTs – MEDLINE sensitive Filter – Cochrane HSSS, 2019] | 15830703 |
| 14 | exp Randomized Controlled Trials as Topic/ or Clinical Trial, Phase II/ or Clinical Trial, Phase III/ or Clinical Trial, Phase IV/ or (equivalence trial or pragmatic clinical trial).pt. or (randomised or randomi#ation? or RCT or placebo$ or ((singl$ or doubl$ or trebl$ or tripl$) adj (mask$ or blind$ or dumm$)) or ((study or trial or CT) adj3 (phase 2 or phase 2a or phase 2b or phase 2c or phase II or phase IIa or phase IIb or phase IIc or phase 3 or phase 3a or phase 3b or phase 3c or phase III or phase IIIa or phase IIIb or phase IIIc or "phase? 2/3" or "phase? II/III" or phase 4 or phase 4a or phase 4b or phase 4c or phase IV or phase IVa or phase IVb or phase IVc or "phase? 3/4" or "phase? III/IV")) or open label$).tw,kw,kf. [PHASE 2-4, OPEN LABEL - ADDITIONAL TERMS TO SUPPLEMENT RCTs FILTER] | 2544014 |
| 15 | Cohort studies/ or comparative study/ or follow-up studies/ or prospective studies/ or risk factors/ or cohort.mp. or compared.mp. or groups.mp. or multivariate.mp. [NON-RANDOMIZED STUDIES – MEDLINE Filter - sensitive, Furlan,2006] | 21592726 |
| 16 | Comparative study/ or Follow-up studies/ or Time factors/ or (preoperat$ or pre operat$).mp. or (chang$ or evaluat$ or reviewed or prospective$ or retrospective$ or baseline or cohort or case series).tw. [OBSERVATIONAL STUDIES – MEDLINE Filter – max specificity, Fraser, 2006] | 26789231 |
| 17 | non-randomized controlled trials as topic/ or controlled before-after studies/ or interrupted time series analysis/ or historically controlled study/ or case-control studies/ or cross-sectional studies/ or observational study/ or (((cohort or concurrent or non-concurrent or incidence or follow-up or followup or longitudinal or prospective or retrospective or nonrandom$ or non-random$ or quasi-random$ or quasi-experiment$ or quasirandom$ or quasiexperiment$ or pretest or posttest or pre-test or post-test or "before after" or CBA or (historical$ adj2 control$) or case-control$ or case-comparison or case-compeer or case-referrent or case-referent or case-base or casecontrol$ or casecomparison or casecompeer or casereferrent or casereferent or casebase or cross-section$ or crosssection$ or prevalence) adj3 (stud$ or design?)) or non-RCT or nRCT or real-world or "real life evidence" or RWE or regist$ or (ITS adj2 (stud$ or design$)) or (interrupted adj2 time adj2 series)).tw,kw,kf. [ADDITIONAL TERMS TO SUPPLEMENT NRS FILTERS] | 6911334 |
| 18 | ("single arm" adj2 (stud$ or design?)).tw,kw,kf. [SINGLE-ARM STUDIES – MEDLINE] | 15006 |
| 19 | 13 or 14 or 15 or 16 or 17 or 18 | 39460875 |
| 20 | (systematic review or systematic literature review or systematic scoping review or systematic narrative review or systematic qualitative review or systematic evidence review or systematic quantitative review or "systematic meta-review" or systematic critical review or systematic mixed studies review or systematic mapping review or systematic cochrane review or "systematic search and review" or systematic integrative review).ti. not comment.pt. not (protocol or protocols).ti. not MEDLINE.st. | 312050 |
| 21 | (1469-493X or 1361-6137).is. and review.pt. | 29351 |
| 22 | systematic review.pt. | 240334 |
| 23 | 20 or 21 or 22 [Ovid Expert Searches: SLR filter 2019] | 562918 |
| 24 | (meta-analy$ or metanaly$ or metaanaly$ or met-analy$).mp,pt. or review.pt. [SLR & MA - modified; Montori, 2004 - Balanced query, sn>sp Filter ] | 6793348 |
| 25 | Network Meta-Analysis/ or ((network adj1 (MA or MAs)) or (NMA or NMAs or MTC or MTCs or MAIC or MAICs or ITC or ITCs or STC or STCs) or indirect$ compar$ or (indirect treatment$ adj1 compar$) or (mixed treatment$ adj1 compar$) or (multiple treatment$ adj1 compar$) or (multi-treatment$ adj1 compar$) or simultaneous$ compar$ or mixed comparison?).tw,kw,kf. [Additional terms for MA, NMA, ITC] | 66145 |
| 26 | (cochrane or health technology assessment or evidence report or systematic reviews).jw. | 69781 |
| 27 | (systematic overview$ or evidence-based review$ or evidence-based overview$ or (evidence adj3 (review$ or overview$ or synthes$)) or meta-review$ or meta-overview$ or meta-synthes$ or metareview$ or metaoverview$ or metasynthes$ or rapid review$ or "review of reviews" or umbrella review? or technology assessment$ or HTA or HTAs).tw,kw,kf. [Additional terms for synonyms for systematic reviews and HTAs based on SLRs] | 230602 |
| 28 | 23 or 24 or 25 or 26 or 27 [SLR & MA FILTERS - Combined] | 7026913 |
| 29 | 19 or 28 | 42731189 |
| 30 | 12 and 29 | 1371 |
| 31 | exp Animals/ not (exp Animals/ and Humans/) [ANIMAL STUDIES ONLY - REMOVE - MEDLINE] | 16825298 |
| 32 | (address or autobiography or bibliography or biography or comment or dictionary or directory or editorial or "expression of concern" or festschrift or historical article or interactive tutorial or lecture or legal case or legislation or news or newspaper article or patient education handout or personal narrative or portrait or video-audio media or webcast or (letter not (letter and randomized controlled trial))).pt. [Opinion publications - Remove -MEDLINE] | 4934519 |
| 33 | 30 not (31 or 32) [ANIMAL STUDIES and OPINION PUBLICATIONS - REMOVED - MEDLINE] | 1093 |
| 34 | limit 33 to yr="2019 -Current" | 767 |
| 35 | 34 use ppez [MEDLINE results] | 122 |
| 36 | multiple myeloma/ or (((myelom$ or leuk?em$) adj4 (multiple or multiplex or plasma-cell? or plasmacell$ or plasmacyt$)) or "myeloma-multiple?" or kahler$ or myelomatos#s or (MM and (myeloma? or leuk?emi$))).ti,ab,kw,kf. [MULTIPLE MYELOMA TERMS] | 185354 |
| 37 | exp hematopoietic stem cell transplantation/ or (((h?ematopoietic or h?emato-poietic) adj3 stem-cell? adj3 (transplant$ or therap$ or transfer$)) or ((HCT? or HSC? or HSCT? or allo-HSC$ or alloHSC$ or auto-HSC$ or autoHSC$) and (transplant$ or therap$ or transfer$ or allogeneic$ or allogenic$ or allo-geneic$ or allo-genic$ or peripheral$ or autologous)) or (transplant$ adj2 (ineligibl$ or "not eligible" or non-eligible or "not qualified" or "not suitable" or "not appropriate" or "not fit" or TIE or NTE)) or (non-transplant$ adj2 (MM or multiple myelom$ or patient? or candidate?))).ti,ab,kw,kf. [HSCT or TRANSPLANT-INELIGIBLE TERMS] | 227304 |
| 38 | 36 and 37 [MM & HSCT TERMS] | 14662 |
| 39 | daratumumab/ or (daratumumab$2 or dalinvi$2 or darasarex$2 or darzalex$2 or hlx-15 or hlx15 or humax-CD38 or humax-CD-38 or jnj-54767414 or jnj54767414 or 945721-28-8 or 4Z63YK6E0E).ti,ab,kw,kf,ot,rn,dq. [DARATUMUMAB TERMS] | 8466 |
| 40 | bortezomib/ or (bortezomib$ or brotezamide$2 or milatib$2 or mylosome$2 or velcad$2 or radiciol$2 or "peptide boronate" or "proscript boronic acid" or pyz-phe-boroleu or bxcl-101 or bxcl101 or hsb-407 or hsb-408 or hsb407 or hsb408 or jnj-26866138 or jnj26866138 or 324b697 or 3mg0 or a18332 or ab01273951-03 or ab01273951-01 or ab01273951-02 or akos015909706 or am81235 or as-15721 or b5741 or bb164258 or bdbm50069989 or brd-k88510285-001-02-0 or c19h25bn4o4 or ccg-268449 or chebi-52717 or chembl325041 or cs-1039 or d03150 or db00188 or dpba or dtxsid3040980 or en300-657180 or gtpl6391 or hb4036 or hy-10227 or mfcd09056737 or mln-341 or mls004774142 or ncgc00168751-01 or ncgc00181022-01 or ncgc00242506-01 or ncgc00242506-02 or ncgc00242506-06 or ncgc00242506-07 or nci60-029010 or nsc756655 or nsc-756655 or q419319 or s1013 or schembl192129 or smr003500787 or sr-01000939863 or sr-01000939863-2 or sw208077-3 or tox21-112630 or tox21-112672 or tox21-112672-1 or z2213886907 or zinc169746649 or nsc-681239 or nsc681239 or mg-341 or mg341 or lpd341 or lpd-341 or ldp-341 or ldp341 or hsdb-7666 or mln-341 or mln341 or ps-341 or ps341 or 341-ps or 179324-69-7 or 197730-97-5 or 444576-08-3 or 1610526-91-4 or 69G8BD63PP).ti,ab,kw,kf,ot,rn,dq. [BORTEZOMIB TERMS] | 54523 |
| 41 | lenalidomide/ or (lenalidomid$ or revimid$2 or revlimid$2 or ladevina$2 or 732L726 or ab01273975-03 or ab01273975-01 or ab01273975-02 or ac-914 or akos005146276 or akos005174869 or albb-015321 or am20050439 or bcp01390 or bcp9000847 or bcpp000186 or bdbm65454 or bl164614 or bp-27972 or c13h13n3o3 or ccg-264781 or chebi-63791 or chembl848 or cs-0125 or d04687 or db00480 or dtxsid8046664 or ec-000-2340 or en300-118706 or f0p408n6v4 or ft-0659651 or ft-0670758 or ft-0670759 or gtpl7331 or hms3654g07 or hms3674c05 or hy-a0003 or imid-5013 or ks-1207 or mfcd07772307 or mls003899194 or ncgc00167491-01 or ncgc00167491-02 or ncgc00167491-03 or ncgc00167491-04 or nsc747972 or nsc-747972 or q-101410 or q425681 or s1029 or sb66166 or schembl1980410 or schembl32978 or smr002529986 or sr-01000883999 or sr-01000883999-1 or stk639603 or sw218084-2 or sy047646 or syp-1512 or syp1512 or tox21-112492 or tox21-112492-1 or z1515385074 or hsdb-8220 or hsdb8220 or cc-5013 or cc5013 or cdc-501 or cdc-5013 or cdc501 or cdc5013 or enmd-0997 or enmd0997 or imid-3 or imid3 or 191732-72-6 or 1243329-97-6 or 202271-91-8 or 874946-00-6 or 443912-14-9 or F0P408N6V4).ti,ab,kw,kf,ot,rn,dq. [LENALIDOMIDE TERMS] | 36089 |
| 42 | dexamethasone/ or (dexamethason$ or adrecort$2 or 064d136 or 1p93 or ab00918428-05 or ab00918428-08 or ab00918428-09 or ab00918428-10 or ac-11056 or adrenocot$2 or aeroseb-d$2 or aeroseb-dex$2 or aflucoson$2 or aflucosone$2 or ai3-50934 or akos005259009 or akos015895509 or alfalyl$2 or amy28815 or anaflogistico$2 or aphtasolon$2 or aphthasolone$2 or apo-dexamethasone$2 or arcodexan$2 or arcodexane$2 or artrosone$2 or auxiron$2 or azium$2 or baycadron$2 or bdbm18207 or bidd-er0494 or bidd-pxr0060 or bidexol$2 or bisu-ds$2 or brd-k38775274-001-02-3 or brd-k38775274-001-06-4 or bspbio-000995 or calonat$2 or ccg-264887 or ccris-7067 or cebedex$2 or cetadexon$2 or chebi-41879 or chembl384467 or colofoam$2 or corsona$2 or corsone$2 or cortastat$2 or cortidex$2 or cortidexason$2 or cortidrona$2 or cortidrone$2 or cortisumman$2 or cs-0626118 or cs-1505 or d00292 or dacortina-fuerte$2 or dacortine-fuerte$2 or dalalone$2 or danasone$2 or db01234 or decacort$2 or decacortin$2 or decadeltosona$2 or decadeltosone$2 or decaderm$2 or decadion$2 or decadran$2 or decadron$2 or decadronal$2 or decadrone$2 or decaesadril$2 or decagel$2 or decaject$2 or decalix$2 or decameth$2 or decamethasone$2 or decasone$2 or decaspray$2 or decasterolone$2 or decdan$2 or decilone$2 or decofluor$2 or dectancyl$2 or dekacort$2 or delladec$2 or deltafluoren$2 or deltafluorene$2 or dergramin$2 or deronil$2 or desacort$2 or desacortone$2 or desadrene$2 or desalark$2 or desameton$2 or desametone$2 or deseronil$2 or desigdron$2 or de-sone-la$2 or dexa-cortisyl$2 or dexa-dabrosan$2 or dexa-korti$2 or dexa-mamallet$2 or dexa-scherosan$2 or dexa-scherozon$2 or dexa-scherozone$2 or dexacen-4$2 or dexachel$2 or dexacidin$2 or dexacort$2 or dexacortal$2 or dexacorten$2 or dexa-cortidelt$2 or dexacortin$2 or dexacortisyl$2 or dexa-cortisyl$2 or dexadabroson$2 or dexadecadrol$2 or dexadeltone$2 or dexadrol$2 or dexafarma$2 or dexagel$2 or dexagen$2 or dexahelvacort$2 or dexakorti$2 or dexalien$2 or dexalocal$2 or dexalona$2 or dexame$2 or dexamecortin$2 or dexameson$2 or dexamesone$2 or dexametason$2 or dexametasone$2 or dexameth$2 or dexamethason$2 or dexamethazon$2 or dexamethazone$2 or dexamethonium$2 or dexamonozon$2 or dexan$2 or dexane$2 or dexano$2 or dexa-p$2 or dexapolcort$2 or dexapos$2 or dexapot$2 or dexaprol$2 or dexascheroson$2 or dexa-scheroson$2 or dexascherozon$2 or dexa-scherozon$2 or dexascherozone$2 or dexa-sine$2 or dexasite$2 or dexason$2 or dexasone$2 or dex-ide$2 or dexinolon$2 or dexinoral$2 or dexionil$2 or dexmethsone$2 or dexona$2 or dexone$2 or dexone$2 or dexpak$2 or dextelan$2 or dextenza$2 or dextrasone$2 or dexycu$2 or dezone$2 or dibasona$2 or dinormon$2 or diodex$2 or doxamethasone$2 or dtxsid3020384 or dxms$2 or einecs-200-003-9 or en300-52607 or esacortene$2 or ex-s1 or exadion$2 or exadione$2 or firmalone$2 or fluormethyl-prednisolone$2 or fluormethylprednisolone$2 or fluormethyl-prednisolon$2 or fluormethylprednisolon$2 or fluoromethylprednisolone$2 or fluoromethyl-prednisolone$2 or fluormone$2 or fluorocort$2 or fluorodelta$2 or fortecortin$2 or gammacorten$2 or gammacortene$2 or grosodexon$2 or grosodexone$2 or gtpl2768 or gtpl3447 or hb2521 or hemady$2 or hexadecadiol$2 or hexadecadrol$2 or hexadiol$2 or hexadrol$2 or hl-dex or hms1792a17 or hms1990a17 or hms2089n13 or hms2235f08 or hms3039l11 or hms3259n11 or hms3403a17 or hsdb 3053 or hy-14648 or hy-14648g or ibi-10090 or isnacort$2 or isoptodex$2 or isopto-dex$2 or isoptomaxidex$2 or isopto-maxidex$2 or isv-305 or isv305 or ks-1451 or ks1451 or lokalison-f$2 or loverine$2 or luxazone$2 or marvidione$2 or maxidex$2 or maxitrol$2 or mediamethasone$2 or megacortin$2 or mephameson$2 or mephamesone$2 or metasolon$2 or metasolone$2 or methazon-ion$2 or methazone-ion$2 or methazonion$2 or methazonione$2 or methylfluorprednisolone$2 or methylfluor-prednisolone$2 or metisone-lafi$2 or mexasone$2 or mexidex$2 or mfcd00064136 or millicorten$2 or millicortenol$2 or mk-125 or mk125 or mls001055412 or mls001332507 or mls001332508 or molmap-000018 or mymethasone$2 or nc00645 or ncgc00091019-01 or ncgc00091019-02 or ncgc00091019-03 or ncgc00091019-04 or ncgc00091019-05 or ncgc00091019-06 or ncgc00091019-07 or ncgc00091019-08 or ncgc00091019-23 or ncgc00257676-01 or nci60-003067 or neoforderx$2 or neofordex$2 or nisomethasona$2 or novocort$2 or nsc-34521 or nsc34521 or ocu-trol$2 or oftan-dexa$2 or opticorten$2 or opticortinol$2 or oradexan$2 or oradexon$2 or oradexone$2 or orgadrone$2 or osurdex$2 or oto-104 or oto104 or ozurdex$2 or pet-derm-iii$2 or phl-dexamethasone$2 or pidexon$2 or policort$2 or posurdex$2 or predni-f-tablinen$2 or predni-f$2 or prednisolon-f$2 or prednisolone-f$2 or prodex$2 or prodexona$2 or prodexone$2 or q-200939 or q422252 or s1322 or sanamethasone$2 or santenson$2 or santeson$2 or sawasone$2 or schembl3774 or sgcut00126 or sk-0503 or sk0503 or smp1-000092 or smr000857119 or smr001227192 or solurex$2 or spectrum5-002019 or spoloven$2 or spt-2101 or spt2101 or sterasone$2 or sunia-sol-d$2 or superprednol$2 or thilodexine$2 or to-000038 or tox21-200122 or triamcimetil$2 or turbinaire$2 or vexamet$2 or visumetazone$2 or visumethazone$2 or zema-pak$2 or zinc3875332 or 23495-06-9 or 1950-02-02 or 1050677-47-8 or 137098-19-2 or 8054-59-9 or 906362-70-7 or 906422-84-2 or 50-02-2 or Z756391748 or 7S5I7G3JQL).ti,ab,kw,kf,ot,rn,dq. [DEXAMETHASONE TERMS] | 301193 |
| 43 | (39 and 41 and 42) or DRd.ti,ab,kw,kf. [DRd TERMS] | 4610 |
| 44 | (40 and 41 and 42) or VRd.ti,ab,kw,kf. [VRd TERMS] | 11044 |
| 45 | 38 and 43 [MM & DRd TERMS] | 438 |
| 46 | 38 and 44 [MM & VRd TERMS] | 1313 |
| 47 | 45 or 46 [MM & DRd or VRd TERMS] | 1409 |
| 48 | Randomized controlled trial/ or Controlled clinical study/ or randomization/ or intermethod comparison/ or double blind procedure/ or human experiment/ or (compare or compared or comparison or trial).ti. or ((evaluated or evaluate or evaluating or assessed or assess) and (compare or compared or comparing or comparison)).ab. or (random$ or placebo or (open adj label) or ((double or single or doubly or singly) adj (blind or blinded or blindly)) or parallel group$1 or (crossover or cross over) or ((assign$ or match or matched or allocation) adj5 (alternate or group$1 or intervention$1 or patient$1 or subject$1 or participant$1)) or (assigned or allocated) or (controlled adj7 (study or design or trial)) or (volunteer or volunteers)).ti,ab. | 11782359 |
| 49 | (Cross-sectional study/ not (randomized controlled trial/ or controlled clinical study/ or controlled study/ or randomi?ed controlled.ti,ab. or control group$1.ti,ab.)) or ((((case adj control$) and random$) not randomi?ed controlled) or (nonrandom$ not random$) or "Random field$" or (random cluster adj3 sampl$)).ti,ab. or (Systematic review not (trial or study)).ti. or ((review.ab. and review.pt.) not trial.ti.) or ("we searched".ab. and (review.ti. or review.pt.)) or ("update review" or (databases adj4 searched)).ab. or ((rat or rats or mouse or mice or swine or porcine or murine or sheep or lambs or pigs or piglets or rabbit or rabbits or cat or cats or dog or dogs or cattle or bovine or monkey or monkeys or trout or marmoset$1).ti. and animal experiment/) or (Animal experiment/ not (human experiment/ or human/)) | 6165531 |
| 50 | 48 not 49 [RCTs – Embase sensitive Filter – Cochrane HSSS, 2019] | 10718496 |
| 51 | phase 2 clinical trial/ or phase 3 clinical trial/ or phase 4 clinical trial/ or (equivalence trial or pragmatic clinical trial).pt. or (randomised or randomi#ation? or RCT or placebo* or ((singl$ or doubl$ or trebl$ or tripl$) adj (mask$ or blind$ or dumm$)) or ((study or trial or CT) adj3 (phase 2 or phase 2a or phase 2b or phase 2c or phase II or phase IIa or phase IIb or phase IIc or phase 3 or phase 3a or phase 3b or phase 3c or phase III or phase IIIa or phase IIIb or phase IIIc or "phase? 2/3" or "phase? II/III" or phase 4 or phase 4a or phase 4b or phase 4c or phase IV or phase IVa or phase IVb or phase IVc or "phase? 3/4" or "phase? III/IV")) or open label$).tw,kw,kf. [PHASE 2-4, OPEN LABEL - ADDITIONAL TERMS TO SUPPLEMENT RCTs FILTER] | 2245496 |
| 52 | Clinical article/ or controlled study/ or major clinical study/ or prospective study/ or cohort.mp. or compared.mp. or groups.mp. or multivariate.mp. [NON-RANDOMIZED STUDIES– Embase Filter - sensitive, Furlan,2006] | 26252988 |
| 53 | Controlled study/ or Treatment outcome/ or Major clinical study/ or Clinical trial/ or (chang$ or evaluat$ or reviewed or baseline or (compare$ or compara$)).tw. [OBSERVATIONAL STUDIES – Embase Filter – max specificity, Fraser, 2006] | 34468489 |
| 54 | exp cohort analysis/ or exp case control study/ or controlled clinical trial/ or pretest posttest control group design/ or static group comparison/ or retrospective study/ or longitudinal study/ or intervention study/ or family study/ or case study/ or time series analysis/ or cross-sectional study/ or comparative study/ or observational study/ or quasi experimental study/ or (((cohort or concurrent or non-concurrent or incidence or follow-up or followup or longitudinal or prospective or retrospective or nonrandom$ or non-random$ or quasi-random$ or quasi-experiment$ or quasirandom$ or quasiexperiment$ or pretest or posttest or pre-test or post-test or "before after" or CBA or (historical$ adj2 control$) or case-control$ or case-comparison or case-compeer or case-referrent or case-referent or case-base or casecontrol$ or casecomparison or casecompeer or casereferrent or casereferent or casebase or cross-section$ or crosssection$ or prevalence) adj3 (stud$ or design?)) or non-RCT or nRCT or real-world or "real life evidence" or RWE or regist$ or (ITS adj2 (stud$ or design$)) or (interrupted adj2 time adj2 series)).tw,kw,kf. [ADDITIONAL TERMS TO SUPPLEMENT NRS FILTERS] | 14665952 |
| 55 | ("single arm" adj2 (stud$ or design?)).tw,kw,kf. [SINGLE-ARM STUDIES – Embase] | 15006 |
| 56 | 50 or 51 or 52 or 53 or 54 or 55 | 43068849 |
| 57 | exp meta analysis/ or ((meta adj analy$) or metaanalys$).mp. or (systematic adj (review? or overview?)).tw. or (cancerlit or cochrane or embase or psychlit or psyclit or psychinfo or psycinfo or cinahl or cinhal or science citation index or bids or reference lists or bibliograph$ or hand-search$ or manual search$ or relevant journals).ab. | 1277306 |
| 58 | (data extraction or selection criteria).ab. and review.pt. | 73419 |
| 59 | 57 or 58 [SLR & MA FILTER - Ovid Expert Searches: SLR filter 2019] | 1289010 |
| 60 | (meta-analy$ or metanaly$ or metaanaly$ or met-analy$).mp. or review.pt. [SLR & MA FILTER - modified and translated; Montori, 2004 - Balanced query, sn>sp Filter ] | 6793348 |
| 61 | network meta-analysis/ or ((network adj1 (MA or MAs)) or (NMA or NMAs or MTC or MTCs or MAIC or MAICs or ITC or ITCs or STC or STCs) or indirect$ compar$ or (indirect treatment$ adj1 compar$) or (mixed treatment$ adj1 compar$) or (multiple treatment$ adj1 compar$) or (multi-treatment$ adj1 compar$) or simultaneous$ compar$ or mixed comparison?).tw,kw,kf. [Additional terms for MA, NMA, ITC] | 66145 |
| 62 | (cochrane or health technology assessment or evidence report or systematic reviews).jw. | 69781 |
| 63 | (systematic overview$ or evidence-based review$ or evidence-based overview$ or (evidence adj3 (review$ or overview$ or synthes$)) or meta-review$ or meta-overview$ or meta-synthes$ or metareview$ or metaoverview$ or metasynthes$ or rapid review$ or "review of reviews" or umbrella review? or technology assessment$ or HTA or HTAs).tw,kw,kf. [Additional terms for synonyms for systematic reviews and HTAs based on SLRs] | 230602 |
| 64 | 59 or 60 or 61 or 62 or 63 [SLR & MA FILTERS - Combined] | 7162238 |
| 65 | 56 or 64 | 46879759 |
| 66 | 47 and 65 | 1314 |
| 67 | (exp animal/ or exp animal experimentation/ or exp animal model/ or exp animal experiment/ or nonhuman/ or exp vertebrate/) not (exp human/ or exp human experimentation/ or exp human experiment/) [ANIMAL STUDIES ONLY - REMOVE - EMBASE] | 12425946 |
| 68 | (editorial or note or short survey or tombstone).pt. or (letter.pt. not randomized controlled trial/) [OPINION PIECES REMOVE - Embase] | 5295391 |
| 69 | 66 not (67 or 68) [ANIMAL STUDIES and OPINION PUBLICATIONS - REMOVED - Embase] | 1257 |
| 70 | limit 69 to yr="2019 -Current" | 749 |
| 71 | conference abstract.pt. | 4794043 |
| 72 | 69 and 71 [CONFERENCE ABSTRACTS ONLY] | 331 |
| 73 | limit 72 to yr="2018 -Current" | 248 |
| 74 | 70 or 73 [Embase results 2019-Current & Conference Abstracts 2018-Current] | 771 |
| 75 | 74 use oemezd [Embase results] | 557 |
| 76 | exp Multiple Myeloma/ or (((myelom$ or leuk?em$) adj4 (multiple or multiplex or plasma-cell? or plasmacell$ or plasmacyt$)) or "myeloma-multiple?" or kahler$ or myelomatos#s or (MM and (myeloma? or leuk?emi$))).ti,ab,kw. [MULTIPLE MYELOMA TERMS] | 184402 |
| 77 | exp Hematopoietic Stem Cell Transplantation/ or (((h?ematopoietic or h?emato-poietic) adj3 stem-cell? adj3 (transplant$ or therap$ or transfer$)) or ((HCT? or HSC? or HSCT? or allo-HSC$ or alloHSC$ or auto-HSC$ or autoHSC$) and (transplant$ or therap$ or transfer$ or allogeneic$ or allogenic$ or allo-geneic$ or allo-genic$ or peripheral$ or autologous)) or (transplant$ adj2 (ineligibl$ or "not eligible" or non-eligible or "not qualified" or "not suitable" or "not appropriate" or "not fit" or TIE or NTE)) or (non-transplant$ adj2 (MM or multiple myelom$ or patient? or candidate?))).ti,ab,kw. [HSCT or TRANSPLANT-INELIGIBLE TERMS] | 225367 |
| 78 | 76 and 77 [MM & HSCT TERMS] | 14480 |
| 79 | (daratumumab$2 or dalinvi$2 or darasarex$2 or darzalex$2 or hlx-15 or hlx15 or humax-CD38 or humax-CD-38 or jnj-54767414 or jnj54767414 or 945721-28-8 or 4Z63YK6E0E).ti,ab,kw. [DARATUMUMAB TERMS] | 6148 |
| 80 | Bortezomib/ or (bortezomib$ or brotezamide$2 or milatib$2 or mylosome$2 or velcad$2 or radiciol$2 or "peptide boronate" or "proscript boronic acid" or pyz-phe-boroleu or bxcl-101 or bxcl101 or hsb-407 or hsb-408 or hsb407 or hsb408 or jnj-26866138 or jnj26866138 or 324b697 or 3mg0 or a18332 or ab01273951-03 or ab01273951-01 or ab01273951-02 or akos015909706 or am81235 or as-15721 or b5741 or bb164258 or bdbm50069989 or brd-k88510285-001-02-0 or c19h25bn4o4 or ccg-268449 or chebi-52717 or chembl325041 or cs-1039 or d03150 or db00188 or dpba or dtxsid3040980 or en300-657180 or gtpl6391 or hb4036 or hy-10227 or mfcd09056737 or mln-341 or mls004774142 or ncgc00168751-01 or ncgc00181022-01 or ncgc00242506-01 or ncgc00242506-02 or ncgc00242506-06 or ncgc00242506-07 or nci60-029010 or nsc756655 or nsc-756655 or q419319 or s1013 or schembl192129 or smr003500787 or sr-01000939863 or sr-01000939863-2 or sw208077-3 or tox21-112630 or tox21-112672 or tox21-112672-1 or z2213886907 or zinc169746649 or nsc-681239 or nsc681239 or mg-341 or mg341 or lpd341 or lpd-341 or ldp-341 or ldp341 or hsdb-7666 or mln-341 or mln341 or ps-341 or ps341 or 341-ps or 179324-69-7 or 197730-97-5 or 444576-08-3 or 1610526-91-4 or 69G8BD63PP).ti,ab,kw. [BORTEZOMIB TERMS] | 54498 |
| 81 | Lenalidomide/ or (lenalidomid$ or revimid$2 or revlimid$2 or ladevina$2 or 732L726 or ab01273975-03 or ab01273975-01 or ab01273975-02 or ac-914 or akos005146276 or akos005174869 or albb-015321 or am20050439 or bcp01390 or bcp9000847 or bcpp000186 or bdbm65454 or bl164614 or bp-27972 or c13h13n3o3 or ccg-264781 or chebi-63791 or chembl848 or cs-0125 or d04687 or db00480 or dtxsid8046664 or ec-000-2340 or en300-118706 or f0p408n6v4 or ft-0659651 or ft-0670758 or ft-0670759 or gtpl7331 or hms3654g07 or hms3674c05 or hy-a0003 or imid-5013 or ks-1207 or mfcd07772307 or mls003899194 or ncgc00167491-01 or ncgc00167491-02 or ncgc00167491-03 or ncgc00167491-04 or nsc747972 or nsc-747972 or q-101410 or q425681 or s1029 or sb66166 or schembl1980410 or schembl32978 or smr002529986 or sr-01000883999 or sr-01000883999-1 or stk639603 or sw218084-2 or sy047646 or syp-1512 or syp1512 or tox21-112492 or tox21-112492-1 or z1515385074 or hsdb-8220 or hsdb8220 or cc-5013 or cc5013 or cdc-501 or cdc-5013 or cdc501 or cdc5013 or enmd-0997 or enmd0997 or imid-3 or imid3 or 191732-72-6 or 1243329-97-6 or 202271-91-8 or 874946-00-6 or 443912-14-9 or F0P408N6V4).ti,ab,kw. [LENALIDOMIDE TERMS] | 36066 |
| 82 | Dexamethasone/ or (dexamethason$ or adrecort$2 or 064d136 or 1p93 or ab00918428-05 or ab00918428-08 or ab00918428-09 or ab00918428-10 or ac-11056 or adrenocot$2 or aeroseb-d$2 or aeroseb-dex$2 or aflucoson$2 or aflucosone$2 or ai3-50934 or akos005259009 or akos015895509 or alfalyl$2 or amy28815 or anaflogistico$2 or aphtasolon$2 or aphthasolone$2 or apo-dexamethasone$2 or arcodexan$2 or arcodexane$2 or artrosone$2 or auxiron$2 or azium$2 or baycadron$2 or bdbm18207 or bidd-er0494 or bidd-pxr0060 or bidexol$2 or bisu-ds$2 or brd-k38775274-001-02-3 or brd-k38775274-001-06-4 or bspbio-000995 or calonat$2 or ccg-264887 or ccris-7067 or cebedex$2 or cetadexon$2 or chebi-41879 or chembl384467 or colofoam$2 or corsona$2 or corsone$2 or cortastat$2 or cortidex$2 or cortidexason$2 or cortidrona$2 or cortidrone$2 or cortisumman$2 or cs-0626118 or cs-1505 or d00292 or dacortina-fuerte$2 or dacortine-fuerte$2 or dalalone$2 or danasone$2 or db01234 or decacort$2 or decacortin$2 or decadeltosona$2 or decadeltosone$2 or decaderm$2 or decadion$2 or decadran$2 or decadron$2 or decadronal$2 or decadrone$2 or decaesadril$2 or decagel$2 or decaject$2 or decalix$2 or decameth$2 or decamethasone$2 or decasone$2 or decaspray$2 or decasterolone$2 or decdan$2 or decilone$2 or decofluor$2 or dectancyl$2 or dekacort$2 or delladec$2 or deltafluoren$2 or deltafluorene$2 or dergramin$2 or deronil$2 or desacort$2 or desacortone$2 or desadrene$2 or desalark$2 or desameton$2 or desametone$2 or deseronil$2 or desigdron$2 or de-sone-la$2 or dexa-cortisyl$2 or dexa-dabrosan$2 or dexa-korti$2 or dexa-mamallet$2 or dexa-scherosan$2 or dexa-scherozon$2 or dexa-scherozone$2 or dexacen-4$2 or dexachel$2 or dexacidin$2 or dexacort$2 or dexacortal$2 or dexacorten$2 or dexa-cortidelt$2 or dexacortin$2 or dexacortisyl$2 or dexa-cortisyl$2 or dexadabroson$2 or dexadecadrol$2 or dexadeltone$2 or dexadrol$2 or dexafarma$2 or dexagel$2 or dexagen$2 or dexahelvacort$2 or dexakorti$2 or dexalien$2 or dexalocal$2 or dexalona$2 or dexame$2 or dexamecortin$2 or dexameson$2 or dexamesone$2 or dexametason$2 or dexametasone$2 or dexameth$2 or dexamethason$2 or dexamethazon$2 or dexamethazone$2 or dexamethonium$2 or dexamonozon$2 or dexan$2 or dexane$2 or dexano$2 or dexa-p$2 or dexapolcort$2 or dexapos$2 or dexapot$2 or dexaprol$2 or dexascheroson$2 or dexa-scheroson$2 or dexascherozon$2 or dexa-scherozon$2 or dexascherozone$2 or dexa-sine$2 or dexasite$2 or dexason$2 or dexasone$2 or dex-ide$2 or dexinolon$2 or dexinoral$2 or dexionil$2 or dexmethsone$2 or dexona$2 or dexone$2 or dexone$2 or dexpak$2 or dextelan$2 or dextenza$2 or dextrasone$2 or dexycu$2 or dezone$2 or dibasona$2 or dinormon$2 or diodex$2 or doxamethasone$2 or dtxsid3020384 or dxms$2 or einecs-200-003-9 or en300-52607 or esacortene$2 or ex-s1 or exadion$2 or exadione$2 or firmalone$2 or fluormethyl-prednisolone$2 or fluormethylprednisolone$2 or fluormethyl-prednisolon$2 or fluormethylprednisolon$2 or fluoromethylprednisolone$2 or fluoromethyl-prednisolone$2 or fluormone$2 or fluorocort$2 or fluorodelta$2 or fortecortin$2 or gammacorten$2 or gammacortene$2 or grosodexon$2 or grosodexone$2 or gtpl2768 or gtpl3447 or hb2521 or hemady$2 or hexadecadiol$2 or hexadecadrol$2 or hexadiol$2 or hexadrol$2 or hl-dex or hms1792a17 or hms1990a17 or hms2089n13 or hms2235f08 or hms3039l11 or hms3259n11 or hms3403a17 or hsdb 3053 or hy-14648 or hy-14648g or ibi-10090 or isnacort$2 or isoptodex$2 or isopto-dex$2 or isoptomaxidex$2 or isopto-maxidex$2 or isv-305 or isv305 or ks-1451 or ks1451 or lokalison-f$2 or loverine$2 or luxazone$2 or marvidione$2 or maxidex$2 or maxitrol$2 or mediamethasone$2 or megacortin$2 or mephameson$2 or mephamesone$2 or metasolon$2 or metasolone$2 or methazon-ion$2 or methazone-ion$2 or methazonion$2 or methazonione$2 or methylfluorprednisolone$2 or methylfluor-prednisolone$2 or metisone-lafi$2 or mexasone$2 or mexidex$2 or mfcd00064136 or millicorten$2 or millicortenol$2 or mk-125 or mk125 or mls001055412 or mls001332507 or mls001332508 or molmap-000018 or mymethasone$2 or nc00645 or ncgc00091019-01 or ncgc00091019-02 or ncgc00091019-03 or ncgc00091019-04 or ncgc00091019-05 or ncgc00091019-06 or ncgc00091019-07 or ncgc00091019-08 or ncgc00091019-23 or ncgc00257676-01 or nci60-003067 or neoforderx$2 or neofordex$2 or nisomethasona$2 or novocort$2 or nsc-34521 or nsc34521 or ocu-trol$2 or oftan-dexa$2 or opticorten$2 or opticortinol$2 or oradexan$2 or oradexon$2 or oradexone$2 or orgadrone$2 or osurdex$2 or oto-104 or oto104 or ozurdex$2 or pet-derm-iii$2 or phl-dexamethasone$2 or pidexon$2 or policort$2 or posurdex$2 or predni-f-tablinen$2 or predni-f$2 or prednisolon-f$2 or prednisolone-f$2 or prodex$2 or prodexona$2 or prodexone$2 or q-200939 or q422252 or s1322 or sanamethasone$2 or santenson$2 or santeson$2 or sawasone$2 or schembl3774 or sgcut00126 or sk-0503 or sk0503 or smp1-000092 or smr000857119 or smr001227192 or solurex$2 or spectrum5-002019 or spoloven$2 or spt-2101 or spt2101 or sterasone$2 or sunia-sol-d$2 or superprednol$2 or thilodexine$2 or to-000038 or tox21-200122 or triamcimetil$2 or turbinaire$2 or vexamet$2 or visumetazone$2 or visumethazone$2 or zema-pak$2 or zinc3875332 or 23495-06-9 or 1950-02-02 or 1050677-47-8 or 137098-19-2 or 8054-59-9 or 906362-70-7 or 906422-84-2 or 50-02-2 or Z756391748 or 7S5I7G3JQL).ti,ab,kw. [DEXAMETHASONE TERMS] | 298260 |
| 83 | (79 and 81 and 82) or DRd.ti,ab,kw. [DRd TERMS] | 3636 |
| 84 | (80 and 81 and 82) or VRd.ti,ab,kw. [VRd TERMS] | 11025 |
| 85 | 78 and 83 [MM & DRd TERMS] | 306 |
| 86 | 78 and 84 [MM & VRd TERMS] | 1298 |
| 87 | 85 or 86 [MM & DRd or VRd TERMS] | 1382 |
| 88 | (editorial or note or comment).pt. or (letter.pt. not randomized controlled trial/) [OPINION PIECES REMOVE - CENTRAL] | 5217895 |
| 89 | 87 not 88 [OPINION PIECES REMOVED - CENTRAL] | 1306 |
| 90 | limit 89 to yr="2019 -Current" | 757 |
| 91 | Conference proceeding.pt. [CONFERENCE ABSTRACTS/PROCEEDINGS] | 221325 |
| 92 | 89 and 91 [CONFERENCE ABSTRACTS ONLY] | 62 |
| 93 | limit 92 to yr="2018 -Current" | 47 |
| 94 | 90 or 93 [CENTRAL results 2019-Current & Conference Abstracts 2018-Current] | 762 |
| 95 | 94 use cctr [CENTRAL results] | 93 |
| 96 | (((myelom$ or leuk?em$) adj4 (multiple or multiplex or plasma-cell? or plasmacell$ or plasmacyt$)) or "myeloma-multiple?" or kahler$ or myelomatos#s or (MM and (myeloma? or leuk?emi$))).ti,ab,kw. [MULTIPLE MYELOMA TERMS] | 147192 |
| 97 | (((h?ematopoietic or h?emato-poietic) adj3 stem-cell? adj3 (transplant$ or therap$ or transfer$)) or ((HCT? or HSC? or HSCT? or allo-HSC$ or alloHSC$ or auto-HSC$ or autoHSC$) and (transplant$ or therap$ or transfer$ or allogeneic$ or allogenic$ or allo-geneic$ or allo-genic$ or peripheral$ or autologous)) or (transplant$ adj2 (ineligibl$ or "not eligible" or non-eligible or "not qualified" or "not suitable" or "not appropriate" or "not fit" or TIE or NTE)) or (non-transplant$ adj2 (MM or multiple myelom$ or patient? or candidate?))).ti,ab,kw. [HSCT or TRANSPLANT-INELIGIBLE TERMS] | 164884 |
| 98 | 96 and 97 [MM & HSCT TERMS] | 7408 |
| 99 | (daratumumab$2 or dalinvi$2 or darasarex$2 or darzalex$2 or hlx-15 or hlx15 or humax-CD38 or humax-CD-38 or jnj-54767414 or jnj54767414 or 945721-28-8 or 4Z63YK6E0E).ti,ab,kw. [DARATUMUMAB TERMS] | 6148 |
| 100 | (bortezomib$ or brotezamide$2 or milatib$2 or mylosome$2 or velcad$2 or radiciol$2 or "peptide boronate" or "proscript boronic acid" or pyz-phe-boroleu or bxcl-101 or bxcl101 or hsb-407 or hsb-408 or hsb407 or hsb408 or jnj-26866138 or jnj26866138 or 324b697 or 3mg0 or a18332 or ab01273951-03 or ab01273951-01 or ab01273951-02 or akos015909706 or am81235 or as-15721 or b5741 or bb164258 or bdbm50069989 or brd-k88510285-001-02-0 or c19h25bn4o4 or ccg-268449 or chebi-52717 or chembl325041 or cs-1039 or d03150 or db00188 or dpba or dtxsid3040980 or en300-657180 or gtpl6391 or hb4036 or hy-10227 or mfcd09056737 or mln-341 or mls004774142 or ncgc00168751-01 or ncgc00181022-01 or ncgc00242506-01 or ncgc00242506-02 or ncgc00242506-06 or ncgc00242506-07 or nci60-029010 or nsc756655 or nsc-756655 or q419319 or s1013 or schembl192129 or smr003500787 or sr-01000939863 or sr-01000939863-2 or sw208077-3 or tox21-112630 or tox21-112672 or tox21-112672-1 or z2213886907 or zinc169746649 or nsc-681239 or nsc681239 or mg-341 or mg341 or lpd341 or lpd-341 or ldp-341 or ldp341 or hsdb-7666 or mln-341 or mln341 or ps-341 or ps341 or 341-ps or 179324-69-7 or 197730-97-5 or 444576-08-3 or 1610526-91-4 or 69G8BD63PP).ti,ab,kw. [BORTEZOMIB TERMS] | 36900 |
| 101 | (lenalidomid$ or revimid$2 or revlimid$2 or ladevina$2 or 732L726 or ab01273975-03 or ab01273975-01 or ab01273975-02 or ac-914 or akos005146276 or akos005174869 or albb-015321 or am20050439 or bcp01390 or bcp9000847 or bcpp000186 or bdbm65454 or bl164614 or bp-27972 or c13h13n3o3 or ccg-264781 or chebi-63791 or chembl848 or cs-0125 or d04687 or db00480 or dtxsid8046664 or ec-000-2340 or en300-118706 or f0p408n6v4 or ft-0659651 or ft-0670758 or ft-0670759 or gtpl7331 or hms3654g07 or hms3674c05 or hy-a0003 or imid-5013 or ks-1207 or mfcd07772307 or mls003899194 or ncgc00167491-01 or ncgc00167491-02 or ncgc00167491-03 or ncgc00167491-04 or nsc747972 or nsc-747972 or q-101410 or q425681 or s1029 or sb66166 or schembl1980410 or schembl32978 or smr002529986 or sr-01000883999 or sr-01000883999-1 or stk639603 or sw218084-2 or sy047646 or syp-1512 or syp1512 or tox21-112492 or tox21-112492-1 or z1515385074 or hsdb-8220 or hsdb8220 or cc-5013 or cc5013 or cdc-501 or cdc-5013 or cdc501 or cdc5013 or enmd-0997 or enmd0997 or imid-3 or imid3 or 191732-72-6 or 1243329-97-6 or 202271-91-8 or 874946-00-6 or 443912-14-9 or F0P408N6V4).ti,ab,kw. [LENALIDOMIDE TERMS] | 24560 |
| 102 | (dexamethason$ or adrecort$2 or 064d136 or 1p93 or ab00918428-05 or ab00918428-08 or ab00918428-09 or ab00918428-10 or ac-11056 or adrenocot$2 or aeroseb-d$2 or aeroseb-dex$2 or aflucoson$2 or aflucosone$2 or ai3-50934 or akos005259009 or akos015895509 or alfalyl$2 or amy28815 or anaflogistico$2 or aphtasolon$2 or aphthasolone$2 or apo-dexamethasone$2 or arcodexan$2 or arcodexane$2 or artrosone$2 or auxiron$2 or azium$2 or baycadron$2 or bdbm18207 or bidd-er0494 or bidd-pxr0060 or bidexol$2 or bisu-ds$2 or brd-k38775274-001-02-3 or brd-k38775274-001-06-4 or bspbio-000995 or calonat$2 or ccg-264887 or ccris-7067 or cebedex$2 or cetadexon$2 or chebi-41879 or chembl384467 or colofoam$2 or corsona$2 or corsone$2 or cortastat$2 or cortidex$2 or cortidexason$2 or cortidrona$2 or cortidrone$2 or cortisumman$2 or cs-0626118 or cs-1505 or d00292 or dacortina-fuerte$2 or dacortine-fuerte$2 or dalalone$2 or danasone$2 or db01234 or decacort$2 or decacortin$2 or decadeltosona$2 or decadeltosone$2 or decaderm$2 or decadion$2 or decadran$2 or decadron$2 or decadronal$2 or decadrone$2 or decaesadril$2 or decagel$2 or decaject$2 or decalix$2 or decameth$2 or decamethasone$2 or decasone$2 or decaspray$2 or decasterolone$2 or decdan$2 or decilone$2 or decofluor$2 or dectancyl$2 or dekacort$2 or delladec$2 or deltafluoren$2 or deltafluorene$2 or dergramin$2 or deronil$2 or desacort$2 or desacortone$2 or desadrene$2 or desalark$2 or desameton$2 or desametone$2 or deseronil$2 or desigdron$2 or de-sone-la$2 or dexa-cortisyl$2 or dexa-dabrosan$2 or dexa-korti$2 or dexa-mamallet$2 or dexa-scherosan$2 or dexa-scherozon$2 or dexa-scherozone$2 or dexacen-4$2 or dexachel$2 or dexacidin$2 or dexacort$2 or dexacortal$2 or dexacorten$2 or dexa-cortidelt$2 or dexacortin$2 or dexacortisyl$2 or dexa-cortisyl$2 or dexadabroson$2 or dexadecadrol$2 or dexadeltone$2 or dexadrol$2 or dexafarma$2 or dexagel$2 or dexagen$2 or dexahelvacort$2 or dexakorti$2 or dexalien$2 or dexalocal$2 or dexalona$2 or dexame$2 or dexamecortin$2 or dexameson$2 or dexamesone$2 or dexametason$2 or dexametasone$2 or dexameth$2 or dexamethason$2 or dexamethazon$2 or dexamethazone$2 or dexamethonium$2 or dexamonozon$2 or dexan$2 or dexane$2 or dexano$2 or dexa-p$2 or dexapolcort$2 or dexapos$2 or dexapot$2 or dexaprol$2 or dexascheroson$2 or dexa-scheroson$2 or dexascherozon$2 or dexa-scherozon$2 or dexascherozone$2 or dexa-sine$2 or dexasite$2 or dexason$2 or dexasone$2 or dex-ide$2 or dexinolon$2 or dexinoral$2 or dexionil$2 or dexmethsone$2 or dexona$2 or dexone$2 or dexone$2 or dexpak$2 or dextelan$2 or dextenza$2 or dextrasone$2 or dexycu$2 or dezone$2 or dibasona$2 or dinormon$2 or diodex$2 or doxamethasone$2 or dtxsid3020384 or dxms$2 or einecs-200-003-9 or en300-52607 or esacortene$2 or ex-s1 or exadion$2 or exadione$2 or firmalone$2 or fluormethyl-prednisolone$2 or fluormethylprednisolone$2 or fluormethyl-prednisolon$2 or fluormethylprednisolon$2 or fluoromethylprednisolone$2 or fluoromethyl-prednisolone$2 or fluormone$2 or fluorocort$2 or fluorodelta$2 or fortecortin$2 or gammacorten$2 or gammacortene$2 or grosodexon$2 or grosodexone$2 or gtpl2768 or gtpl3447 or hb2521 or hemady$2 or hexadecadiol$2 or hexadecadrol$2 or hexadiol$2 or hexadrol$2 or hl-dex or hms1792a17 or hms1990a17 or hms2089n13 or hms2235f08 or hms3039l11 or hms3259n11 or hms3403a17 or hsdb 3053 or hy-14648 or hy-14648g or ibi-10090 or isnacort$2 or isoptodex$2 or isopto-dex$2 or isoptomaxidex$2 or isopto-maxidex$2 or isv-305 or isv305 or ks-1451 or ks1451 or lokalison-f$2 or loverine$2 or luxazone$2 or marvidione$2 or maxidex$2 or maxitrol$2 or mediamethasone$2 or megacortin$2 or mephameson$2 or mephamesone$2 or metasolon$2 or metasolone$2 or methazon-ion$2 or methazone-ion$2 or methazonion$2 or methazonione$2 or methylfluorprednisolone$2 or methylfluor-prednisolone$2 or metisone-lafi$2 or mexasone$2 or mexidex$2 or mfcd00064136 or millicorten$2 or millicortenol$2 or mk-125 or mk125 or mls001055412 or mls001332507 or mls001332508 or molmap-000018 or mymethasone$2 or nc00645 or ncgc00091019-01 or ncgc00091019-02 or ncgc00091019-03 or ncgc00091019-04 or ncgc00091019-05 or ncgc00091019-06 or ncgc00091019-07 or ncgc00091019-08 or ncgc00091019-23 or ncgc00257676-01 or nci60-003067 or neoforderx$2 or neofordex$2 or nisomethasona$2 or novocort$2 or nsc-34521 or nsc34521 or ocu-trol$2 or oftan-dexa$2 or opticorten$2 or opticortinol$2 or oradexan$2 or oradexon$2 or oradexone$2 or orgadrone$2 or osurdex$2 or oto-104 or oto104 or ozurdex$2 or pet-derm-iii$2 or phl-dexamethasone$2 or pidexon$2 or policort$2 or posurdex$2 or predni-f-tablinen$2 or predni-f$2 or prednisolon-f$2 or prednisolone-f$2 or prodex$2 or prodexona$2 or prodexone$2 or q-200939 or q422252 or s1322 or sanamethasone$2 or santenson$2 or santeson$2 or sawasone$2 or schembl3774 or sgcut00126 or sk-0503 or sk0503 or smp1-000092 or smr000857119 or smr001227192 or solurex$2 or spectrum5-002019 or spoloven$2 or spt-2101 or spt2101 or sterasone$2 or sunia-sol-d$2 or superprednol$2 or thilodexine$2 or to-000038 or tox21-200122 or triamcimetil$2 or turbinaire$2 or vexamet$2 or visumetazone$2 or visumethazone$2 or zema-pak$2 or zinc3875332 or 23495-06-9 or 1950-02-02 or 1050677-47-8 or 137098-19-2 or 8054-59-9 or 906362-70-7 or 906422-84-2 or 50-02-2 or Z756391748 or 7S5I7G3JQL).ti,ab,kw. [DEXAMETHASONE TERMS] | 178849 |
| 103 | (99 and 101 and 102) or DRd.ti,ab,kw. [DRd TERMS] | 3314 |
| 104 | (100 and 101 and 102) or VRd.ti,ab,kw. [VRd TERMS] | 6152 |
| 105 | 98 and 103 [MM & DRd TERMS] | 238 |
| 106 | 98 and 104 [MM & VRd TERMS] | 595 |
| 107 | 105 or 106 [MM & DRd or VRd TERMS] | 674 |
| 108 | limit 107 to yr="2019 -Current" | 462 |
| 109 | 108 use coch [CDSR results] | 0 |
| 110 | 35 or 75 or 95 or 109 [All databases] | 772 |
| **111** | **remove duplicates from 110 [All databases results deduplicated]** | **570** |

Supplemental Table 2: Risk of Bias

| **Study name** | **TAURUS**[**^26^**](#_ENREF_26) | **MAIA vs SWOG S0777**[**^27^**](#_ENREF_27) | **PEGASUS**[**^25^**](#_ENREF_25) |
| --- | --- | --- | --- |
| Was the cohort recruited in an acceptable way? | Yes | Not clear | Yes |
| Was the exposure accurately measured to minimise bias? | Not clear | Yes | Not clear |
| Was the outcome accurately measured to minimise bias? | Not clear | Yes | Not clear |
| Have the authors identified all important confounding factors? | Not clear | Not clear | No |
| Have the authors taken account of the confounding factors in the design and/or analysis? | Yes | Yes | Yes |
| Was the follow-up of patients complete? | Yes | Yes | Yes |
| How precise (for example, in terms of confidence interval) are the results? | Yes | Yes | Yes |
| **Overall risk of bias** | **Moderate risk** | **Moderate risk** | **High risk** |

Note: green highlighting denotes a low risk of bias, yellow highlighting denotes a moderate risk of bias and red highlighting denotes a high risk of bias.
